# Sustainable development goals: a call for future internal medicine

**DOI:** 10.1007/s11739-025-03941-3

**Published:** 2025-04-11

**Authors:** Maria Regina Ferrando, Elisa Proietti, Maria Demontis, Fabrizio Montecucco, Livia Pisciotta

**Affiliations:** 1 Department of Internal Medicine and Medical Specialties, University of Genoa, Viale Benedetto XV 6, 16132 Genoa, Italy; 2 Ospedale Policlinico San Martino IRCCS, Largo Rosanna Benzi 10, 16132 Genoa, Italy; 3 Department of Internal Medicine, University of Genoa, viale Benedetto XV 6, 16132 Genoa, Italy; 4 IRCCS Ospedale Policlinico San Martino, Genoa - Italian Cardiovascular Network, Largo Rosanna Benzi 10, 16132 Genoa, Italy

**Keywords:** SDGs, Internal medicine, Healthcare, Patient-centered approach, Sustainability

## Abstract

2030 Agenda Sustainable Development Goals (SDGs) achievement is the main challenge in order to design a sustainable future society. SDG 3 ensure healthy lives and promote well-being for all at all ages, focusing on prevention and treatment of communicable diseases (CDs) and non-communicable diseases (NCDs). Moreover, access to care, affordable drugs, and vaccines is crucial to improve quality of life leaving no one behind. Internal medicine can answer to the United Nations (UN) universal call by taking action both on infectious disease burden and on NCDs challenge, enabling prevention strategies and treatments, providing new drugs and ensuring accessible care for all. Furthermore, thanks to internist holistic and patient-centered approach, together with a multidisciplinary strategy, misallocation of economic and human resources, and medical errors due to healthcare fragmentation could be avoided. Nonetheless, a syndemic approach taking into account economic, social, and environmental dimensions of health will become fundamental for healthcare systems design. In this framework, internal medicine will be crucial giving a comprehensive patient-centered perspective, coordinating multidisciplinary team interventions, and contributing to sustainable healthcare systems.

**Graphical abstract:**

Prevalence of major global causes of death by groups in 2021 according to Wolrd Health Organization World Health Statistics 2024 [12]; Internal Medicine characteristics; Internal Medicine-related SDGs summary; Future challenges for Internal Medicine 
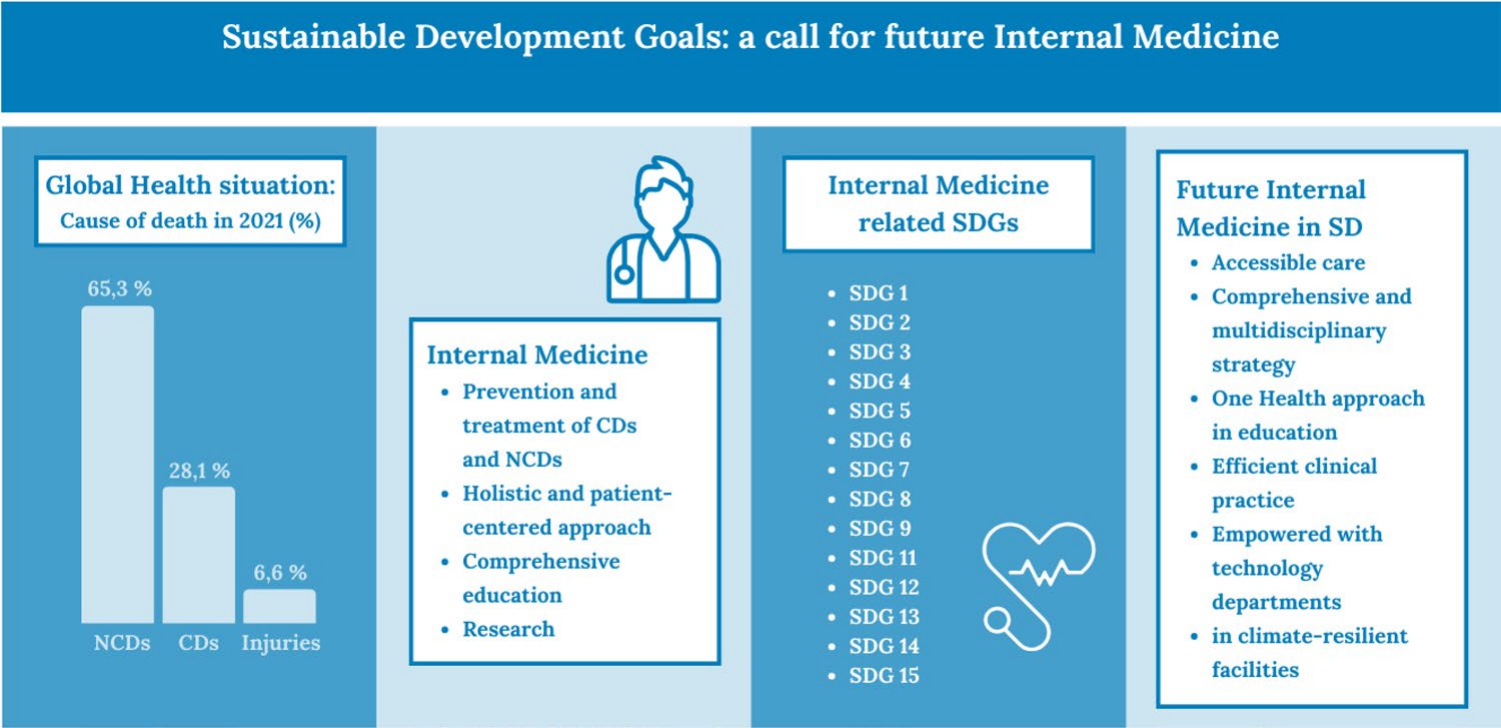

## Introduction

A sustainable development is pivotal aiming to design a sustainable future society which guarantees healthy lives, decent work for all, and shared prosperity respecting our planet. This is stated in the 2030 Agenda, a resolution of the United Nations (UN) adopted in 2015, addressed to all countries and stakeholders. The 2030 Agenda is an action plan detailed in 17 Goals and 169 Targets to fulfill in order to make Sustainable Development happen toward 2030 and beyond. Leaving no one behind is crucial in a Sustainable Development framework, ensuring equitable and universal access to nutritious foods, adequate healthcare, and social protection, preserving physical, mental, and social well-being. Therefore, improving public health and enhancing sustainability awareness, considering environmental, economic, and social dimensions, is key to achieving the sustainable development goals (SDGs) [1]. Among the SDGs, there are many interconnected either directly or indirectly to healthcare issues. Particularly, Goal 3 is dedicated to “ensure healthy lives and promote well-being for all at all ages”, focusing on communicable diseases (CDs) and non-communicable diseases (NCDs) prevention and treatment, access to care, and affordable drugs and vaccines, to improve quality of life for all [2]. Although all sectors are taking action to optimize their practices leading to encouraging advancements, it is necessary to strengthen our commitment [3]. Regarding health-related goal progress, the situation is mixed showing overall positive trend, even considering the great impact of COVID-19 pandemic [3]. On the one hand, the main health-related targets are close to be met. As an example, AIDs mortality has been halved, thanks to effective treatments from 2010 to 2023. On the other hand, 381 million people were pushed into extreme poverty in 2019 due to out-of-pocket payments for health, and universal health coverage (UHC) achievement is still a long way off [4]. Internal medicine can answer to the 2030 Agenda universal call by taking action on infectious disease burden and on NCDs challenge, enabling prevention strategies and treatments, providing new drugs, ensuring accessible care, enhancing equity of healthcare services, optimizing human and material resources, taking into account the environmental impact. In this regard, despite the empowerment of the healthcare systems of the last decades led to better health outcomes, their environmental impact is not negligible [5]. Globally healthcare systems are responsible for 4.6% of greenhouse gas emissions and contribute to the emissions of particulate matter, hazardous waste and other environmental pollutants [6, 7]. In the SDGs era, high-quality health systems are required to optimize resources, enhancing resilience and increasing equitability, without neglecting the environmental footprint, according to The Lancet Global Health Commission [8]. Climate-resilient and environmentally sustainable healthcare facility goals should focus on sustainability of healthcare workforces, accurate management of outputs and appropriate infrastructures provided with innovative technologies [9]. Since internal medicine is a core specialty in the healthcare systems dealing with clinical practice, medical education and research through a holistic and patient-centered approach, internists could be the suitable professionals to fight climate change in the healthcare sector [10, 11]. The current review will point out the correlation of internal medicine to several SDGs, clarifying the current situation on health worldwide, exploring health-related SDGs, and highlighting internal medicine role, challenges, and limitations in achieving a sustainable future society.

## Global situation on health

The continuing trend of increasing life expectancy and decreasing mortality due to improvements in living conditions, income, education, and medicine, is well known [12]. Globally, life expectancy at birth has increased by about 7.4 years from 1990 to 2017. In the less-developed countries, this increase is largely due to the sharp decline in perinatal and infant mortality, whereas in high-income countries, it is mainly due to a decrease in mortality among the elderly [13]. Overall, the causes of death can be grouped into three categories: CDs (including infectious, parasitic, maternal, perinatal, and nutritional conditions), NCDs (chronic conditions), and injuries [14]. In the last decades, the onset of an epidemiologic transition has occurred showing a rise in NCDs worldwide. Nevertheless, CDs still cause significant morbidity and mortality, especially in low-income countries [15]. As a matter of fact, CDs deaths globally accounted for 28.1% in 2021 and lower respiratory tract infections represented the world deadliest CD, temporarily redirecting the trend due to COVID-19 pandemic [16], while NCDs and injuries respectively accounted for 65.3% and around 7% of the main causes of death [14]. NCDs are pathologic conditions closely linked to unhealthy lifestyles and aging, such as cancer, metabolic disorders like diabetes or obesity, cardiovascular diseases, degenerative neurologic diseases, lung diseases and chronic diseases in general [17]. In 2021, 7 out of the 10 leading causes of death were NCDs affecting especially older age groups, leading these individuals to multimorbidity and frailty conditions. [18, 19]. Cardiovascular diseases accounted for most deaths, 17.9 million people per year, followed by cancer (9.3 million), chronic respiratory disease (4.1 million) and diabetes (2.0 million, including kidney deaths caused by diabetes) [14]. Despite global efforts to stop the growing epidemic of metabolic diseases, great disparities remain between low-income, middle-income and high-income countries. According to the Global Burden of Disease (GBD), the onset of metabolic disorders is mainly linked to lifestyle risk factors, such as tobacco use, physical inactivity, harmful alcohol consumption and unhealthy diets. [20]. Furthermore, many environmental risk factors contribute to NCDs [14]. In addition, social risk factors must be considered. Vulnerable and socially disadvantaged people get sick and die earlier than people of higher social status due to several factors including limited access to healthcare services. The Study on global Ageing and adult health (SAGE), highlighted that actual health coverage was insufficient in the included countries, showing that many elderly people either gave up or underutilized health services, or ultimately incurred unaffordable healthcare costs [21]. Furthermore, this study revealed a huge gap in access to medicines and health technologies between high and low-income countries, empathizing the need for innovation and local production to improve the equitable distribution of health products [22]. Since 2015, the percentage of healthcare facilities with a basic set of essential medicines available and affordable (SDG indicator 3.b.3) has been estimated for 17 countries in the WHO region, with a median value of 8% [23]. In 2023, only 0.4 billion more people benefited from UHC and 0.6 billion were better protected from health issues. The most significant improvements in average coverage of essential healthcare services have been observed in the field of infectious diseases, but progress are also occurring on NCD treatment coverage [24].

Role of internal medicine in improving the quality of health status and contribution to the achievement of goals at preventive, therapeutic, clinical, and translational level.

**Fig.1:**
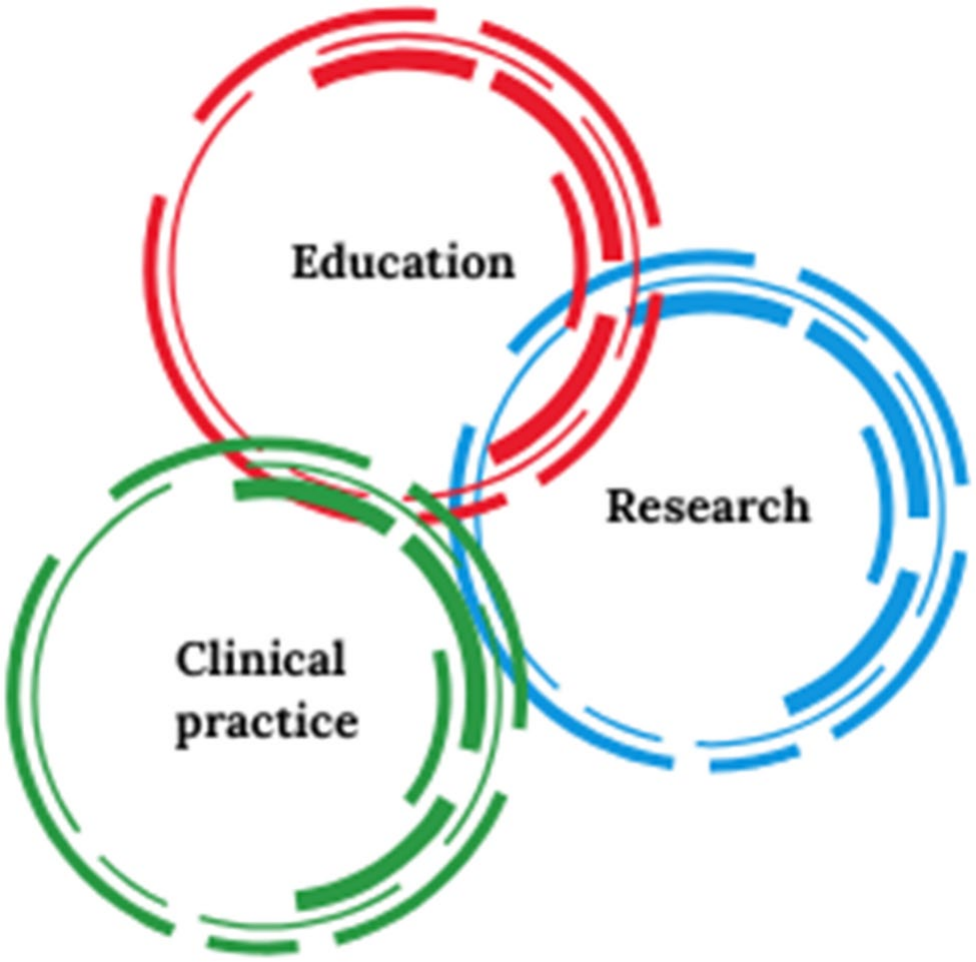
The three pillars of Internal Medicine: medical education, clinical practice and research through a holistic approach [9]

In Italy, according to Ministerial Decree No. 68 of 4 February 2015, the specialty schools in the healthcare area belong to three categories: surgical, medical and clinical services. Although internal medicine is included in the medical area, its practice and structure are variable across countries [25, 26]. Despite being one of the oldest branches of medicine, internal medicine, as shown in Fig. 1, represents the convergence of three cornerstones of modern medicine: research, medical training and clinical practice [11]. Internists are particularly interested in the search for the causes of the diseases, maintaining a comprehensive approach in the management of patients, assessing not only individual symptoms but also the complex interactions between different organs and systems [27]. Indeed, internal medicine can be defined as a patient-centered specialty, focusing on the patient suffering from a pathology instead on the pathology itself [28]. The holistic and patient-centered approach is crucial, allowing internists to address NCDs, multimorbidity conditions and common infectious diseases, as well as tackling new and unexpected medical challenges [18]. Internal medicine is also fundamental for medical student education, providing a wide background and enabling inclusion in care settings [29].

Nevertheless, internal medicine plays a pivotal role in healthcare, improving the overall quality of life, which is key to achieving SDGs toward 2030. Indeed, SDG 3 addresses human well-being considering many factors concerning internal medicine sphere of action, especially CDs and NCDs issues. On the one hand, target 3.3 aims to end CD epidemics, such as AIDS, tuberculosis, hepatitis, neglected tropical diseases and water-borne diseases by 2030 [2]. Over the decades, internists could adapt to multiple scenarios, fighting in the front line of diverse infectious emergencies such as COVID-19 pandemic, thanks to their high versatility and a comprehensive expertise [11]. In this field, internal medicine is a pillar for healthcare all over the world, especially in low-income countries, where CDs were 8 out of 10 leading causes of death in 2021 [16]. On the other hand, target 3.4 focuses on the impact of NCDs on healthy life expectancy, strongly requiring the implementation of a strategy based on prevention and treatment to lower by one-third premature mortality from NCDs [2]. Internists work on prevention and treatment of NCDs drawing a comprehensive clinical picture, often referring to patients presenting multiple chronic conditions since almost 3 out of 4 elderly suffer from multimorbidity as well as 1 out of 4 adults [30]. Moreover, to fight CDs and NCDs, research on vaccines and medications is key as highlighted in Sect. 3.b of the 2030 Agenda [1]. In this framework, internal medicine clinical research plays a fundamental role in developing affordable solutions making healthcare accessible to all. Adults suffering from multimorbidity are the major users of healthcare services in adulthood accounting for more than two-third of healthcare spending due to fragmentation of care [30]. Indeed, internist patient-centered approach is needed in order to avoid resource misallocation and to prevent medical errors leading to increased mortality [31, 32]. Furthermore, a year-long ward-wide observational study conducted in the Unit of General Medicine and Advanced Care at IRCCS Ospedale San Raffaele in Milan (Internal Medicine Department) showed that patients were exposed to high risks of mortality, prolonged hospitalization and development of nosocomial infections, related not only to pre-existing pathologic conditions but also to baseline assistance factors, such as insufficient number of physicians involved in patient care. [33]. On the one hand, under-manning healthcare systems have been already related with negative outcome indicators, suggesting that an increase in human resources is necessary to respond to the growing demand of assistance, especially in hospital settings dealing with multimorbidity and elderly, like Internal Medicine departments [34, 35]. On the other hand, overloading of human resources, stressful work environments and underpayment, due to staff shortage and saving policies, is associated with poor work conditions affecting healthcare professional well-being. [36, 37]. Empowering internal medicine departments could lead to improve employment and decent work conditions, resulting in efficiency of the healthcare systems and aligning with SDG 8.

**Fig.2:**
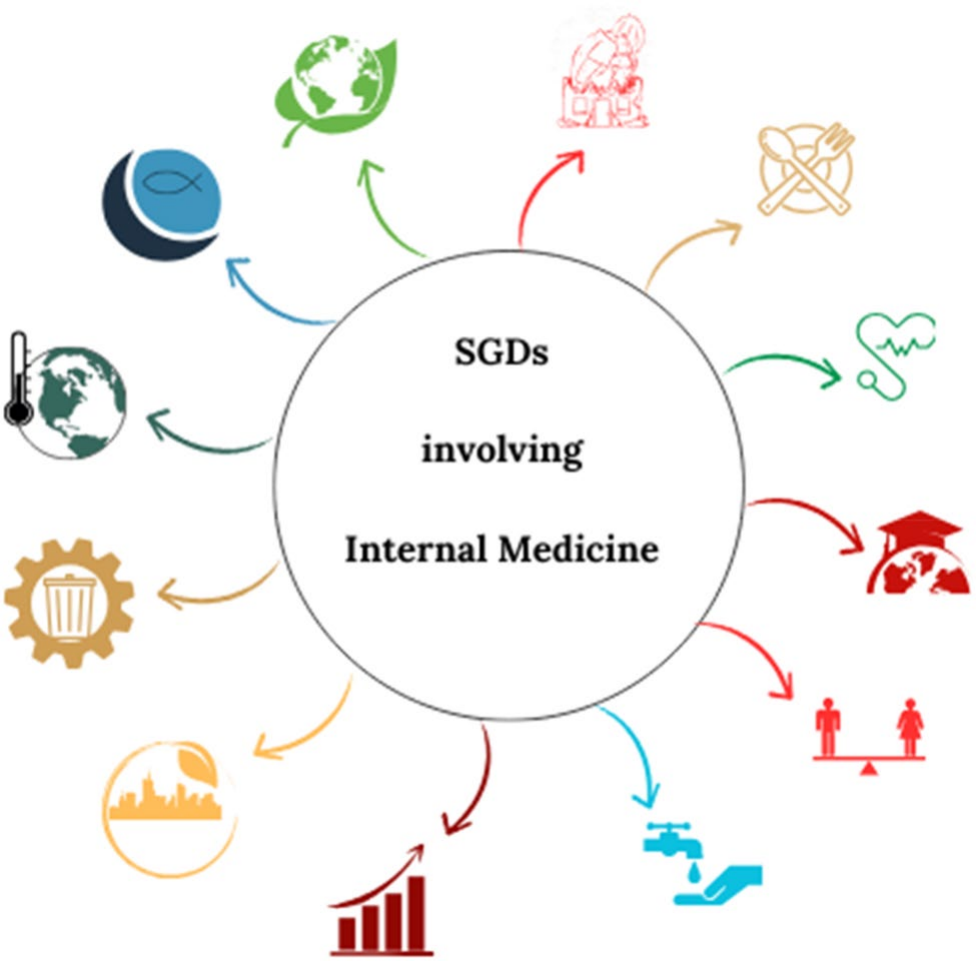
Main SDGs mentioned in the text that are involved in Internal Medicine; Starting from the top right in clockwork direction: “No poverty”, “Zero Hunger”, “Good health and well-being”, “Quality education”, “Gender equality”, “Clean water and sanitation”, “Decent work and economic growth”, “Sustainable cities and communities”, “Responsible consumption and production”, “Climate action”, “Life below water”, and “Life on land” [6];[8];[9]

Many more SDGs are related to health and well-being in their broadest sense, and therefore to Internal Medicine, as outlined in Fig. 2 [1]. Starting from the SDG 2 “Zero hunger” aiming to eradicate not only hunger but all forms of malnutrition, ensuring sufficient nutritious, safe and accessible food for all [1]. Considering malnutrition both as undernutrition and overweight, it is frequently leading to diet-related NCDs [38]. Recently, an increasing number of communities, especially in low- and middle-income countries, suffers both from stunting and from overweight, due to a rapid global nutrition transition. Even the same individual can be exposed to different forms of malnutrition over distinct stages of life, directly experiencing the double burden of malnutrition (DBM) with severe long-term effects (metabolic disorders, chronic inflammation, gut microbiome imbalance) [39]. As a matter of fact, access to quality food and nutritional support are essential in prevention strategies aiming to reduce dietary risk factors and to guarantee a high degree of well-being tackling CDs and NCDs [40, 41]. Additional risk factors contribute to CDs and NCDs development including different environmental conditions, such as air pollution, inappropriate use of chemicals, unhealthy environments, harmful agricultural practices, and unsafe water sources. Indeed, exposure to air pollution increases the risk of adverse health effects, including stroke, ischemic heart disease, lung cancer and chronic obstructive pulmonary disease and acute infections of the lower respiratory tract [14]. These topics are discussed in multiple SDGs, such as SDG 2 nutrition and sustainable agriculture, SDG 3 well-being and hazardous chemicals and air, water and soil pollution and contamination, SDG 6 water and sanitation management, SDG 7 sustainable energy, SDG 9 sustainable and resilient infrastructures, SDG 11 inclusive and sustainable urbanization, SDG 12 sustainable consumption and production patterns, SDGs 14 and 15 sustainable uses of marine and terrestrial ecosystems [1]. Therefore, the integration of healthcare systems, especially internal medicine departments, into SDG efforts is urgent.

Inadequate access to quality water services is a major problem in healthcare facilities in less-developed regions, having implications for sanitation. Availability of sustainable water and sanitation, proper use of chemicals, and proper disposal of healthcare waste are essential for quality care and prevention of infectious diseases. WHO with the United Nations International Children’s Emergency Fund (UNICEF), Member States and partners are actively responding to these issues, trying to promote environmentally sustainable healthcare services through safe water and sewerage management, clean energy, proper waste disposal, and sustainable goods supply [42]. Access to sustainable energy supporting basic services, such as lighting, communications, refrigeration, diagnostic, and medical devices, is also critical to fulfill several SDGs in healthcare facilities [43, 44]. Low-energy efficient buildings, equipment and processes contribute to fuel waste and increases air pollution. Empowering energy efficiency, switching to clean and renewable energy sources, healthcare sector can reduce the environmental footprint while enhancing public health by reducing hospital admissions and treatment for chronic diseases due to pollution, such as asthma, lung and heart disease [9].

Within sustainable healthcare models, remote assistance methods can be acknowledged as a genuine tool for delivering healthcare services. Telemedicine is a clear example of how access to care can be achieved while pursuing SDGs, ensuring coverage of peripheral areas and patients with mobility limitation assistance using remote technologies [45, 46]. Furthermore, it can be displayed as a valuable resource in achieving UHC, overcoming not only financial barriers but also logistical and geographic issues [47]. Multiple examples of evidence-based applications of telemedicine in the field of Internal Medicine can be found in literature, including follow-up projects started by patients suffering from rheumatic diseases [48], remote consultations for patients living in areas with mobility issues [49], and home care support in the elderly [50]. Moreover, telemedicine has proven to be crucial in emergency situations, where immediate action is lifesaving, like in case of stroke [51]. Telemonitoring of chronic conditions, such as chronic heart failure and arrhythmia, and of infectious conditions, is effective [52, 53]. This innovation can be considered as a protective strategy both for patients, occurring to medical centers, and for healthcare professionals, successfully applied during COVID-19 pandemic [53]. The elderly would particularly benefit from telemedicine as continuous monitoring of vital parameters can slow down the progression or exacerbation of chronic conditions [54]. Furthermore, telemedicine seems to mitigate depression, especially for patients living alone, creating a sense of community [55]. As a result, speaking about benefits for patients undergoing therapy, telemedicine is a tool to better customize treatment interventions, to remote monitor real-time clinical parameters, to offer a coordinated multidisciplinary support and to manage regular follow-ups, delivering a personalized quality service straight to patient home. Furthermore, telemedicine enables self-management improving patient quality of life, by enhancing awareness of their own health condition, undergoing autonomous follow-up requests and providing more timely and more targeted care support [56]. In this framework, proof of successful integration of telemedicine in Internal Medicine comes from a case study conducted at the Regional Centre for Pediatric Diabetes of the Giannina Gaslini Institute in Genoa, focused on the pediatric and young adult population with type 1 diabetes. The study assessed the satisfaction of patients and their families with the use of telemedicine in disease management. Results show telemedicine facilitated communication between patient families and healthcare professionals, feeling more comfortable with the tele visits and perceiving more attention and support. [57]. As mentioned before, telemedicine decreases travel costs and mobility difficulties due to limited infrastructure or to patients’ condition by reducing the onsite consultations [58, 59]. From the environmental sustainability point of view, telemedicine offers a valid strategy in pursuing SDG 13, especially applied to internal medicine framework, where follow-up is frequently required for patients affected by NCDs and multimorbidity. According to a “green” position paper, recently drafted by United European Gastroenterology, improving strategies for greener healthcare, such as digitization and telemedicine, is the way forward to reduce carbon footprint [60]. The provision of virtual consultations via telemedicine by a healthcare professional is estimated to reduce emissions by 128 kg CO_2_-eq per patient [61]. The environmental benefits are mainly due to the reduction of traveling greenhouse gas emissions [60]. Decreasing unnecessary medical procedures, like endoscopies, is also crucial to pursuit a sustainable model, reducing carbon footprint [62]. Endoscopy is the third producer of hazardous waste in healthcare settings with 3.09 kg/day/bed, surpassed only by intensive care and anesthetics, respectively 3.37 kg/day/bed and 5.96 kg/day/bed [63, 64]. From a nephrology perspective, instead, it is crucial to develop water-saving and waste-saving methods. Tarrass et al. found that dialysis wastewater could be upcycled as land fertilizer, converting 95% of PO_4_^3−^ and 23% of the NH^3+^ [65]. Circular practices in dialysis are estimated to have the potential to decrease the carbon footprint by a third [66].

Several scientific societies, including the European Federation of Internal Medicine, published a position paper calling for greater involvement of healthcare professionals fighting against climate change, environmental degradation and its consequences on human health [67]. Indeed, the duties of future healthcare professionals include not only patient care but also community well-being, which depends on the ecosystem balance that is increasingly threatened [68]. Mitigating the healthcare sector environmental footprint requires collaborative efforts by all stakeholders and institutions and must be considered a policy priority. In this framework, integrating the One Health Approach into education and training of health professionals is critical for developing the proper skills to address this issue. The inclusion of the SDGs in internist curriculum could raise awareness on climate change effects on human health, enabling best practice implementation [69] Nonetheless, health educational programs addressed to general population could be broadcasted through telemedicine systems, disseminating healthy lifestyles and preventive behaviors, easily reaching a wide audience including people living in rural areas (SDG 4) [70].

SDG 5 focuses on achieving gender equality and women empowerment [1]. Internal medicine, like other healthcare disciplines, is affected by gender inequality. Gender disparities are especially found in the leadership positions of academic medicine and particularly in internal medicine as highlighted by R.M. Hanna and colleagues conducting a retrospective observational study of Internal Medicine ground rounds at three US academic. Despite nowadays women and men graduate in the same proportion from medical school, female chair of departments represents only the 18% [71]. Furthermore, speaking about female patients, gender inequality becomes a risk factor for women health, which is threatened by not only a biologic difference in susceptibilities to diseases, disability, and injuries, but also by discriminatory prejudice of the healthcare professionals and biases in health systems, practices and research [72].

Another key challenge concerning healthcare systems and Internal Medicine is to give access to UHC, moving toward Sustainable Development. The UHC concept is based on the principle that all individuals and communities should have access to quality essential healthcare services across the full spectrum of care, without major financial barriers [14]. This not only contributes to improve health outcomes (SDG 3) but also plays a central role in other SDGs including economic growth and job creation (SDG 8), gender equality (SDG 5), education (SDG 4), nutrition (SDG 2) and poverty reduction (SDG 1) [1].

In conclusion, internal medicine is related to several SDGs, whether directly or indirectly, as highlighted in Fig. 2, intersecting with poverty reduction (SDG1), nutrition issues (SDG 2), quality education (SDG 4), gender equity (SDG 5), water sanitation and management (SDG 6), sustainable energy (SDG 7), efficient systems for economic growth and decent work (SDG 8), sustainable and resilient infrastructures (SDG 9), inclusive and sustainable urbanization (SDG 11), sustainable consumption and production patterns (SDG 12), climate change actions (SDG 13), sustainable use of marine and terrestrial ecosystems (SDGs 14, 15) and of course good health and well-being (SDG 3) [1].

Future internal Medicine in the framework of the SDGs: limitations, challenges, and recommendations.

Future internal medicine could help achieve the established SDGs related to healthcare enhancing prevention, treatments and accessibility to care while improving the efficiency of health systems. Currently, progress on the health-related goals of the SDGs is uncertain. Over 53 SDG health indicators, 32 have numerical targets and most of them show a trend in the right direction in spite of difficulties [3].

Several limitations slow down the achievement of the SDG targets. As already mentioned, principal obstacles are related to financial constraints, workforce shortages, and obsolete low efficiency facilities and procedures [37, 42, 43]. Other limitations include practices which enhance gender inequities both in academic and in patient care settings [71, 72], and the lack of planetary health education in the internist curriculum [67]. Future challenges for internal medicine include implementing a more structured action at preventive and therapeutic level, leveraging their distinctive holistic and patient-centered approach combined with a multidisciplinary strategy, intercepting all conditions that lead to risk of multimorbidity and frailty before they become extremely complex and therefore have a high impact on the resources [73]. Internist practice should aim to reduce the most impactful procedures to strictly necessary, such as endoscopy, and to adopt innovative technologies, like telemedicine, moving first steps toward ensuring sustainability and optimization in the near future [46, 62]. Nonetheless, a syndemic approach, which considers economic, social and environmental dimensions of healthcare, must become a strong driver in internal medicine decision-making, in international guidelines establishment, and, of course, in healthcare facilities design [74]. Ultimately, internist professional profile seems to be the most desirable in the coordination of multidisciplinary teams and in sustainability management in future climate-resilient and environmentally sustainable healthcare systems [11].

To overcome limitations and challenges, recommendations must be issued to address interventions. As already pointed out, scientific societies and academies worldwide have already drawn up some recommendations, such as the position paper of the European Federation of Internal Medicine and the recommendations of the Spanish-Portuguese Internal Medicine services [10, 67]. Currently, a shared Sustainable Clinical Practice Guideline for Italian Internal Medicine is necessary in order to set a structured and responsive workflow. The document should consider the use of more efficient tools and procedures, which could reduce costs and improve departmental performance. Furthermore, a national plan must be elaborated for the reallocation of existing resources in terms of funds and workforce, focusing on multidisciplinary teams, overcoming financial constraints and workforce shortage from a long-term perspective. Another key consideration of the national plan should concern the energy efficiency of healthcare facilities, giving concrete recommendations based on WHO guidelines [9]. Sustainability knowhow, including environmental, social and economic facets, will lay the foundations of future Internal Medicine practices, enabling internists to respond to respond to climate change progress and consequences [8]. Therefore, empowered internal medicine departments will be fundamental in climate-resilient and environmentally sustainable healthcare facilities. Future healthcare systems should be based on sufficient trained healthcare professionals with ethical involvement in sustainable clinical practices and output management, working in sustainable infrastructures supplied with proper instruments and processes [7]. Nevertheless, moving toward gender equity in leadership positions of academic and clinical Internal Medicine must be a priority.

Establishing specific indicators and benchmarks to assess internal medicine contribution to SDGs is essential to measure the advancements of the field. Assessment of environmental and social performance is particularly hard because of multiple factors like long time periods, high-level uncertainty, difficult quantification. [75]. Life cycle assessment is a validated method already implemented in multiple fields including healthcare, but a healthcare-specific LCA must be developed with proper metrics and benchmarks [76]. Globally, common indicators are already applied to healthcare settings, such as greenhouse gas emissions, waste, air pollution, travel, and energy use [77]. Specific metrics should be defined for internal medicine departments, such as bed days, high environmental footprint practices (i.e., endoscopy and dialysis), tele visits, and solid waste [78].

As suited in the 2030 Agenda and stressed in the above recommendations, public institutions and stakeholders must work together strengthening the means of implementation and revitalizing the Global Partnership for Sustainable Development [1]. As well as primary care organizations and public health institutions must coordinate with clinical professionals in a synergetic way in order to provide sustainable and decent healthcare leaving no one behind [6].

## Conclusion

Achieving SDGs worldwide is an urgent task. The sustainability of healthcare systems is a key point of the 2030 Agenda, outlining the need for sustainable essential healthcare services for all. In this context, the integration of internal medicine into SDG efforts could play a pivotal role, thanks to its holistic and patient-centered approach.

Despite multiple limitations persisting, the first actionable steps must include researching sustainable and accessible preventive and therapeutic strategies best suited to patients, optimizing the use of human and planetary resources. The enforcement of shared sustainable policies, particularly a Sustainable Clinical Practice Guideline for Italian Internal Medicine and a national plan on resource reallocation, must be a priority, enhancing the deployment of cost-effective strategies, innovative technologies, such as telemedicine, and modern infrastructures. Ultimately, the One Health approach has to be included in internist education, positioning them at the leading edge of the fight against climate change.
